# Assessment of medium and large‐sized mammals and their behavioral response toward anthropogenic activities in Jorgo‐Wato Protected Forest, Western Ethiopia

**DOI:** 10.1002/ece3.8529

**Published:** 2022-02-11

**Authors:** Mosissa Geleta Erena

**Affiliations:** ^1^ Department of Biology Wollega University Nekemte Ethiopia

**Keywords:** camera trap, Ethiopia, Jorgo‐Wato Protected Forest, large mammals

## Abstract

Medium and large‐sized mammals of Jorgo‐Wato Protected Forest have not yet been documented though the forest established before four decades. Hence, this study aims to document medium and large mammals and the behavioral responses of selected mammals toward anthropogenic activities in the study area. The study was conducted from February 2015 to June 2016, encompassing the wet and dry seasons. Data were collected mainly through camera traps, indirect and direct evidence. The study revealed about 23 medium and large‐sized mammals that belong to seven orders namely *Bovidae*, *Carnivora*, *Primates*, *Rodentia*, *Tubulidentata*, *Lagomorpha*, and *Hyracoidea*. *Papio anubis*, *C*. *guereza*, and *C*. *aethiops* were the most abundant large mammals in JWPF. Because of high anthropogenic activities, African buffalo shifted its activity period from diurnal into crepuscular and nocturnal. African buffalo traveled longer distances during the wet season (mean = 14.33 km, SD = 1.25 km) than during the dry season (mean = 9.00 km, SD = 2.16 km). This could be due to the fact that the local people were less likely to go to the forest for resource exploitation during the wet season as they are fully engaged in agricultural activities. However, low agricultural activities during the dry season allow the local people to extract resources and involve in bushmeat hunting which could limit the movement of mammals to their refugia. African buffalo preferred to rest on and adjacent to a gravel road (22.1%) in the forest, followed by on open rocky hilltops (14.7%) at night time, but rest in the bottomland thicket vegetation during the dry daytime. Regardless of high human pressure in the area, this study has revealed a good number of medium and large‐sized mammals that could be used as baseline information to design a sound conservation and management action plan of large mammals and their habitat in Jorgo‐Wato Protected Forest.

## Graphical abstract

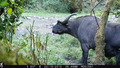

## 1 INTRODUCTION

Mammals are important for the proper functioning of an ecosystem. They play a fundamental role in nutrient cycling (Doughty et al., 2016), recruitment of plants (Snyder et al., 2006), pollination, monitoring the structure and composition of vegetation, and seed dispersal (Jordano et al., 2007). They are also important in fulfilling the needs of humans such as cloth, food, and spiritual values (Boesch et al., 2017). However, mammals are severely affected by habitat loss, overexploitation, invasive species, and climate change (Pacifici et al., 2017). As a result, the extinction of animals in protected areas may affect ecosystem processes in ways that we do not yet comprehend (Boddicker et al., 2002). Therefore, it is imperative to document and monitor mammalian species in and around protected areas to plan on their future conservation and management activities (Nichols & Williams, 2006). The presence or absence of mammals, their distribution, and abundance in different areas can be assessed by various methods (Wilson et al., 1996). Most of the methods developed to survey larger mammals inhabiting open savanna or grassland habitats have been easily applied. In contrast to open habitats, investigating medium and large mammals inhibited in tropical forest habitats is difficult (Andreka et al., 1999). Hence, it is particularly challenging to locate, count, and monitor mammals in tropical forests. This could force researchers to use flexible methods to assess and monitor mammal communities in and around protected areas (Boddicker et al., 2002).

Observations of mammals in the tropical rainforest are difficult because of the thick forest, high canopies, and nocturnal activities of most animals. For such animals, indirect evidence such as footmarks, droppings, sound, and feeding remains is used to verify presence (Burton et al., 2015; Wilson et al., 1996) and used to develop indices of presence and abundance of mammals. A camera trap is a cost‐effective technique used to monitor secretive mammalian species in forested ecosystems (Burton et al., 2015). It is less invasive, time‐consuming, and cheaper than other methods to survey and estimate mammalian species in inaccessible areas (Burton et al., 2015; Cutler & Swann, 1999). Camera traps have been used to quantify the presence and relative abundance of rainforest mammals (Burton et al., 2015; Gast & Stevenson, 2020; Martins et al., 2007). Cameras also have been used by researchers to estimate population density and relative abundance of wildlife species (Burton et al., 2015; Nielsen & McCollough, 2009). It is also used to assess the feeding ecology and activity patterns of mammals (Frey et al., 2017; Springer et al., 2011).

Wildlife species respond to anthropogenic activities that range from behavioral to distributional changes. These changes may depend on the type, intensity, and frequency of anthropogenic activities (Gaynor et al., 2018). Anthropogenic activities are the main cause of the disturbance of large mammals (Darimont et al., 2015). As result, many species of mammals showed a strong fear response to anthropogenetic activities (Clinchy et al., 2016; Smith et al., 2017). Fear created due to anthropogenic activities is known to affect the behavioral and activity patterns of mammals (Suraci et al., 2019). The intensity of anthropogenic activities varies based on season, weekdays (Monday to Friday), and weekends (Saturday and Sunday). Human recreation can negatively affect wildlife, particularly on weekends when human activity is highest (González et al., 2006; Nix et al., 2018). Moreover, the local people commonly collect resources from protected areas during the dry season due to poor agricultural activity, which may confine mammalian activity to their local refugia (González et al., 2006; Perona et al., 2019). On the other hand, the local people participate in agricultural and other livelihood activities throughout the rainy season and on weekdays. This could reduce the impact of human‐induced stresses on mammalian activity patterns (Erena et al., 2020). Such temporal change in human–animal interactions can cause changes in wildlife behavior, such as increased stress, missing foraging opportunities, lower reproductive success, avoidance of certain areas, and higher mortality (Longshore et al., 2013; Martin & Réale, 2008; Simmonds & Keay, 1997).

Diurnal species are sensitive to increased human activities on weekends and weekdays due to greater temporal variation and activity overlap with humans (Longshore et al., 2013; Roy et al., 2014). This may alter the behaviors of mammals to adapt to increased human activities. Continuous human–wildlife interaction may lead to increased stress in wildlife, decrease foraging opportunities, reduce reproductive success, increased mortality, and avoidance of certain habitats by animals (Longshore et al., 2013; Martin & Réale, 2008). It also reduces the activity of individuals, increased avoidance of areas used by humans, altered behaviors, and reduced fitness (Tadesse & Kotler, 2012; Tarjuelo et al., 2015). Recently, increased anthropogenic activities in protected areas have become a global conservation concern as it poses a severe impact mostly on large vertebrate taxa (Monti et al., 2018; Spaul & Heath, 2017). Mitigating the negative impacts of anthropogenic activities on mammals becomes a challenging task to conserve wildlife in protected areas (Krausman et al., 2008).

Understanding wildlife movements and habitat use are critical for species conservation and management on a landscape scale (Allen & Singh, 2016). Movement data can be used to evaluate the response of animals toward anthropogenically disturbed habitats as means of behavioral adaptions and survival (Carbone et al., 2005). Distance traveled by animals can be linked to energy use, risk avoidance, resource use, and the extent of anthropogenic disturbances (Fahrig, 2007). Tracking distance traveled by mammals is very important to address the various issues related to anthropogenic disturbances. Distance traveled by mammals in protected areas is correlated with the different weekdays, weekends, and seasons because humans' access to protected areas for recreation and resource harvest varied temporally (Noonan et al., 2019).

**FIGURE 1:**
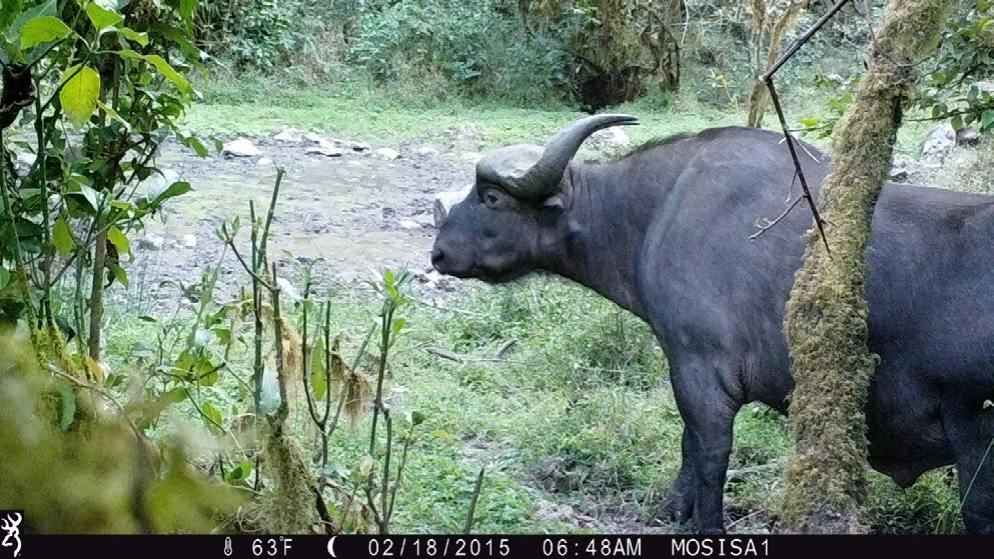
A bull of African buffalo in Jorgo‐Wato Protected Forest

Though Jorgo‐Wato Protected Forest (JWPF) has been established before four decades, its medium and large‐sized mammal species compositions and their response to anthropogenic activities have not yet been studied. This study also aimed to address on the behavioral responses of African buffalo toward human‐induced pressure since the animal shifted its habitat from its ancestral savanna wooded grassland of the adjacent Dhidhessa‐Dabana Valley into completely forested habitat of Jorgo‐Wato Protected Forest. Therefore, this study also aims to examine activity periods of common mammalian species captured by camera traps, and the effects of weekends, weekdays, and seasons on movements of African buffalo in the area (Figure 1).

## 2 MATERIALS AND METHODS

### 2.1 Study area

**FIGURE 2:**
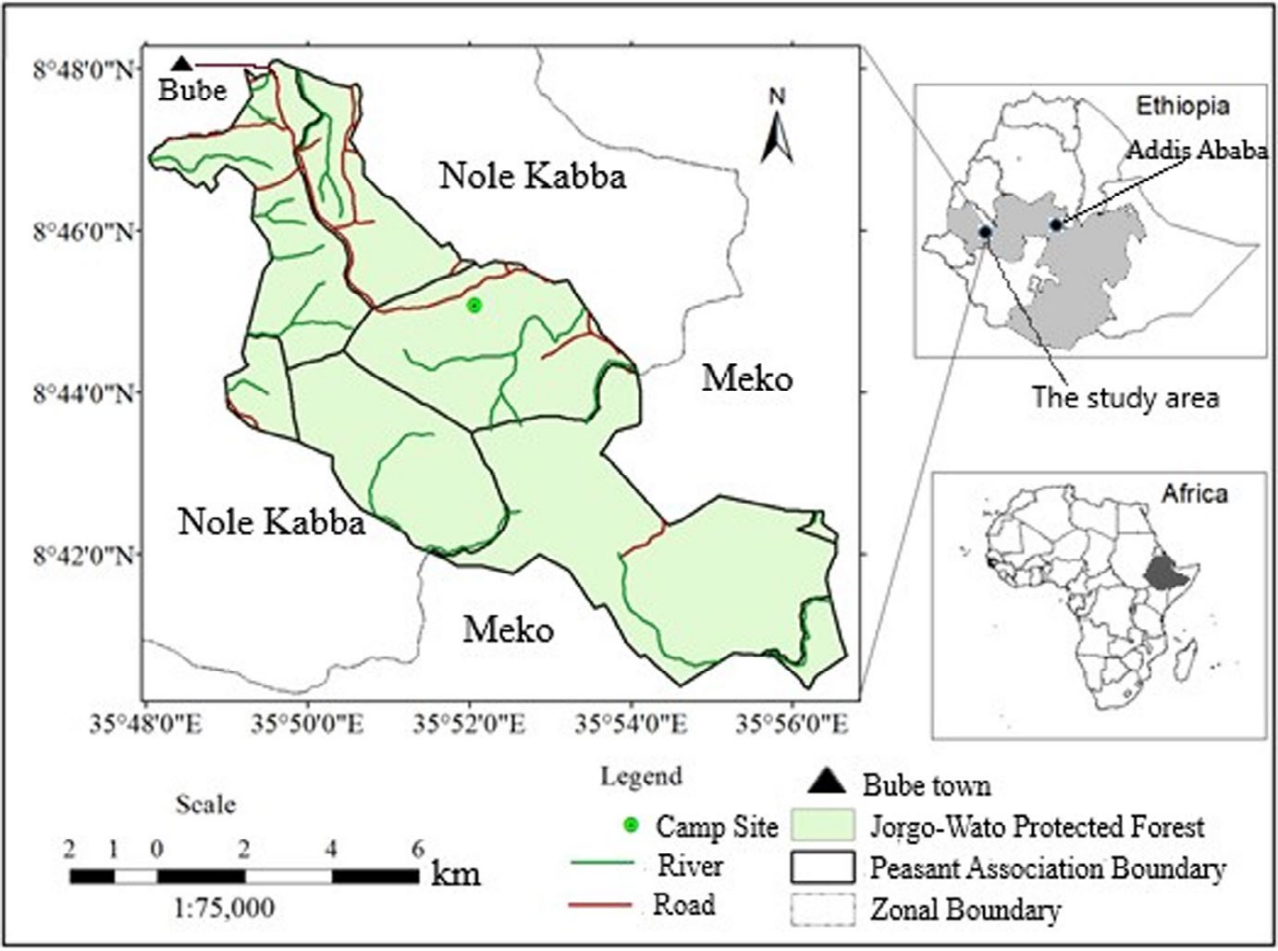
Map of the study area

Jorgo‐Wato Protected Forest (JWPF) is situated between 8°40′20″–8°48′06″N and 35°48′01″–35°56′40″E, about 509 km west of Addis Ababa (Figure 2). It was proposed as one of the top National Forest Priority Areas in Ethiopia in 1976. It had a total area of 19,875 ha in 1988 but is currently reduced to 8,503.49 ha because of human‐induced pressure. Anthropogenic factors that are threatening JWPF are deforestation, lodging trees for timber and beehive preparation, a coffee plantation in the forest, livestock grazing, debarking trees for beehive preparation, and collection of construction materials (Erena et al., 2019). Currently, JWPF was administered by Oromia Forest and Wildlife Enterprise (OFWE), which gives special emphasis to the plantation forest as it is harvested for immediate commercial purposes. The forest is surrounded by six Farmer Associations. These include Harbu Abba Gada, Siba Silassie, Siba Dalo, Asgori Sora, Siba Dalo, and Wato Golbe. The local communities surrounding the forest lead their life through subsistence agriculture and livestock farming.

### 2.2 Methods

**FIGURE 3:**
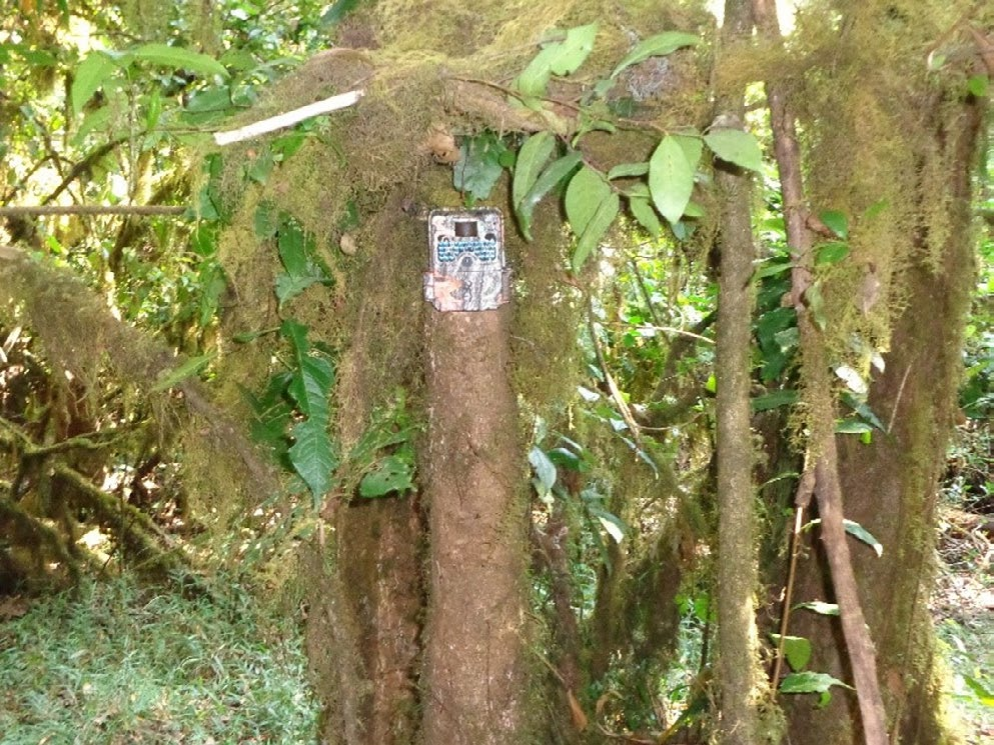
A camera trap fixed at 1 m height during the study period

This study has focused on the medium and large‐sized mammal community composition of the study area. Medium mammals are those whose body weight is between 2 and 5 kg, and large‐sized mammals are those over 5 kg body mass (Njoroge et al., 2009). Indirect and direct evidence and camera traps were employed to assess medium and large‐sized mammal species, their activity patterns, and responses of African buffalo (the largest mammal in the area) to anthropogenetic activities in JWPF. During January, April, and May 2017, 8 HC500 HYPERFIRE camera traps were installed and stationed along common animal trails, salt licks, water holes, and other clear areas where mammal pathways were evidenced. Camera stations were changed and rotated weekly for a total of 56 days for each camera. All camera traps placed at different stations were about 5 km far from each other to make an independent sampling location. At each station, a camera trap was set at the height of about 1 m above the ground and positioned slightly downward (Figure 3). All cameras were adjusted to take three photographs per trigger with an interval of one second between pictures. Only one out of the three photographs captured during each trigger was considered for the estimation of relative abundance and activity patterns of mammals. If the same mammal was assumed to be captured more than three times at a time, the remaining photographs were rejected.

Camera traps were supplied by alkaline batteries and worked 24 h a day recording date and time of each photograph. Camera traps were checked at an interval of two days early in the morning. No bait was used to attract mammals toward the camera trap. In addition to the camera trap, indirect evidence (footprints, droppings, burrows) was also used to collect data along 49 strip transects. During all transects walked, direct observation and indirect evidence such as footprints, burrowing, and droppings were recorded. All fieldworks were carried out between February 2015 and June 2016. Assessment of mammals using a camera trap was carried out during the dry (January 2016) and the wet (April and May 2016) seasons. Species identification was done by examining photographs generated from camera traps and with the assistance of local field guides for indirect evidence. Activity periods for mammals captured by camera trap were classified as diurnal and nocturnal by adopting a method employed by Gómez et al. (2005). Species were classified as diurnal if more than 90% of observations were in the day (6:30 am−7:30 pm) and the remaining 10% in the night, as nocturnal (7:30 pm–5:30 am) if 90% of observations were in the dark and the remaining 10% were in the day or crepuscular (5:30–7:30 am and 5:30–7:30 pm) if 50% of observations made during the crepuscular phase. Moreover, habitat association of medium and large‐sized mammals was inferred from both direct and indirect signs of evidence that show occurrences of mammals assuming that the recorded signs were proportional to their distribution and habitat use (Macleod et al., 1996; Shrestha et al., 2005).

Human‐induced pressure was a serious problem for large mammals in protected areas. African buffalo is one of the largest mammals threatened by anthropogenic activities in the study area. Distance moved by African buffalo during different periods was recorded by backtracking herds of African buffalo using GPS. Distance traveled was quantified by summing the straight‐line displacement between discretely sampled locations (Rowcliffe et al., 2012; Sennhenn‐Reulen et al., 2017) generated from GPS and pulled for weekdays, weekends, and seasons. Besides, the typical resting sites of African buffalo were also recorded along the track as diurnal (based on trampled plants, footmarks, and when observed in the afternoon tracking) and nocturnal (when new resting sites are observed in the morning tracking) to infer their resting preferences in JWPF.

### 2.3 Data analysis

Relative abundance for each species was calculated by summing up the number of individuals recorded in all transects using Microsoft excel 2016. The abundance of medium and large‐sized mammals was computed as the total number of individual species/total number of species observed in the sampled habitat multiplied by a hundred. The frequency of occurrence of medium and large‐sized mammals was described as common (C), frequent (F), occasional (O), and rare (R) (Zerihun et al., (2012). Moreover, the conservation status of mammals identified from JWPF was described as vulnerable and least concerned as per the IUCN Red List of 2008. Differences in the distribution of signs attributed to mammal observations in different habitats and African buffalo resting preferences were tested by chi‐square test of association.

## 3 RESULTS

### 3.1 Species composition of mammals

Twenty‐three medium and large‐sized mammals were recorded in Jorgo‐Wato Protected Forest using a camera trap, direct and indirect evidence (Table 1). Among these, 6 mammal species were evidenced by a total of 464 photographs captured by a camera trap during the study periods. The recorded mammals were categorized into seven orders which include the following: the order *Bovidae*, *Carnivora*, *Primates*, *Rodentia*, *Tubulidentata*, *Lagomorpha*, and *Hyracoidea*. Among all orders, the maximum species were recorded for the order *Carnivora* (8 species), followed by the order *Bovidae* (6 species) and *Primates* (5 species). The order *Hyracoidea* was represented by two species, whereas one species was recorded each for the order *Rodentia*, *Tubulidentata*, and *Lagomorpha*. Most of the recorded mammals were detected by indirect evidence such as droppings and footprints. Except for nonhuman primates, all the detected mammals were rarely seen during the daytime in both seasons.

**TABLE 1:** Mammalian species recorded through camera trap, direct and indirect evidences in Jorgo‐Wato Protected Forest (F = Foot prints; S = Seen; P = Photographs; D = Droppings; B = Borrows, * = Community information)

Order	Species	Common name	Signs recorded
*Bovidae*	*Syncerus caffer*	African buffalo	S, F, P
	*Traglaphus scriptus*	Bushbuck	S, P, D
	*Sylvicapra grimmia*	Common duiker	S, P, D
	*Potamochoerus larvatus*	Bush pig	S, D
	*Hylochoerus meinertzhageni*	Giant forest hog	S, D
	*Phacochoerus africanus*	Warthog	S, D
*Carnivora*	*Crocuta crocuta*	Spotted hyaena	P, D, F
	*Canis aureus*	Common jackal	*
	*Civettictis civetta*	African civet	P, D
	*Panthera pardus*	Leopard	D
	*Felis caracal*	Caracal	S, P
	*Felis sylvestris*	African wild cat	S
	*Leptailurus serval*	Serval cat	S
	*Helogale parvula*	Common dwarf mongooses	P
*Primates*	*Papio anubis*	Olive baboon	S, D, P
	*Colobus guereza*	Colobus monkey	S
	*Chlorocebus aethiops*	Grivet monkey	S
	*Cercopithecus mitis*	Blue monkey	S, P
*Rodentia*	*Histrix cristata*	Crested porcupine	D
*Tubulidentata*	*Orycteropus afer*	Aardvark	B
*Lagomorpha*	*Lepus starcki*	Stark's hare	S
*Hyracoidea*	*Procavia capensis*	Rock hyrax	S
	*Hetrohyrax brucei*	Bush hyrax	S

### 3.2 Relative abundance and conservation status of mammals

During this study period, a total of 877 individual mammals were recorded in JWPF. Among these, Olive baboons were the most abundant comprising 21.55% of the recorded individual mammals followed by Colobus monkey (18.81%) and Grivet monkey (6.42%), respectively. Besides, rocky hyrax (0.34%) and caracal (0.34%) were the least abundant mammalian species followed by a leopard (0.46%) (Table 2).

**TABLE 2:** Number of individual mammal species and their relative abundance in the study area

Family	Species name	Local name	Number of individuals recorded	Relative Abundance (%)
*Bovidae*	*Syncerus caffer*	African buffalo	68	7.75
	*Traglaphus scriptus*	Bushbuck	35	3.99
	*Sylvicapra grimmia*	Common duiker	18	2.05
	*Potamochoerus larvatus*	Bush pig	59	6.73
	*Hylochoerus meinertzhageni*	Giant forest hog	14	1.6
	*Phacochoerus africanus*	Warthog	18	2.05
*Carnivora*	*Crocuta crocuta*	Spotted hyaena	23	2.62
	*Canis aureus*	Common jackal	5	0.57
	*Civettictis civetta*	African civet	8	0.91
	*Panthera pardus*	Leopard	4	0.46
	*Felis caracal*	Caracal	3	0.34
	*Felis sylvestris*	African wild cat	6	0.68
	*Leptailurus serval*	Serval cat	7	0.80
	*Helogale parvula*	Common dwarf mongooses	6	0.68
*Primates*	*Papio anubis*	Olive baboon	189	21.55
	*Colobus guereza*	Colobus monkey	165	18.81
	*Chlorocebus aethiops*	Grivet monkey	144	16.42
	*Cercopithecus mitis*	Blue monkey	57	6.52
*Rodentia*	*Histrix cristata*	Crested porcupine	16	1.82
*Tubulidentata*	*Orycteropus afer*	Aardvark	10	1.14
*Lagomorpha*	*Lepus starcki*	Stark's hare	8	0.91
*Hyracoidea*	*Procavia capensis*	Rock hyrax	3	0.34
	*Hetrohyrax brucei*	Bush hyrax	11	1.3
		Total	877	100

Out of 23 species of mammals recorded in JWPF, 3 species (13.04%) were common, 4 species (17.39%) were frequent, 12 species (52.17%) were occasional, and 4 species (17.39%) were rare. Similarly, among the recorded mammalian species, about 91.30% were categorized as least concern and the remaining 8.70% species were categorized as vulnerable (Table 3).

**TABLE 3:** Frequency of occurrences of mammals recorded during the wet and dry seasons in Jorgo‐Wato Protected Forest

Species name	Local name	Occurrence category	IUCN conservation status
*Syncerus caffer*	African buffalo	Occasional	Least concern
*Traglaphus scriptus*	Common Bushbuck	Occasional	Least concern
*Sylvicapra grimmia*	Common duiker	Occasional	Least concern
*Potamochoerus larvatus*	Bushpig	Frequent	Least concern
*Hylochoerus meinertzhageni*	Giant forest hog	Occasional	Least concern
*Phacochoerus africanus*	Warthog	Occasional	Least concern
*Crocuta crocuta*	Spotted hyaena	Frequent	Least concern
*Canis aureus*	Common jackal	Occasional	Least concern
*Civettictis civetta*	African civet	Occasional	Least concern
*Panthera pardus*	Leopard	Rare	Vulnerable
*Felis caracal*	Caracal	Rare	Least concern
*Felis sylvestris*	African wild cat	Occasional	Least concern
*Felis serval*	Serval cat	Rare	Least concern
*Helogale parvula*	Common dwarf mongooses	Occasional	Least concern
*Papio anubis*	Olive baboon	Common	Least concern
*Colobus guereza*	Colobus monkey	Common	Least concern
*Chlorocebus aethiops*	Grivet monkey	Common	Least concern
*Cercopithecus mitis*	Blue monkey	Frequent	Vulnerable
*Histrix cristata*	Crested porcupine	Occasional	Least concern
*Orycteropus afer*	Aardvark	Occasional	Least concern
*Lepus starcki*	Stark's hare	Occasional	Least concern
*Procavia capensis*	Rock hyrax	Rare	Least concern
*Hetrohyrax brucei*	Bush hyrax	Frequent	Least concern

### 3.3 Activity periods of selected mammals

**FIGURE 4:**
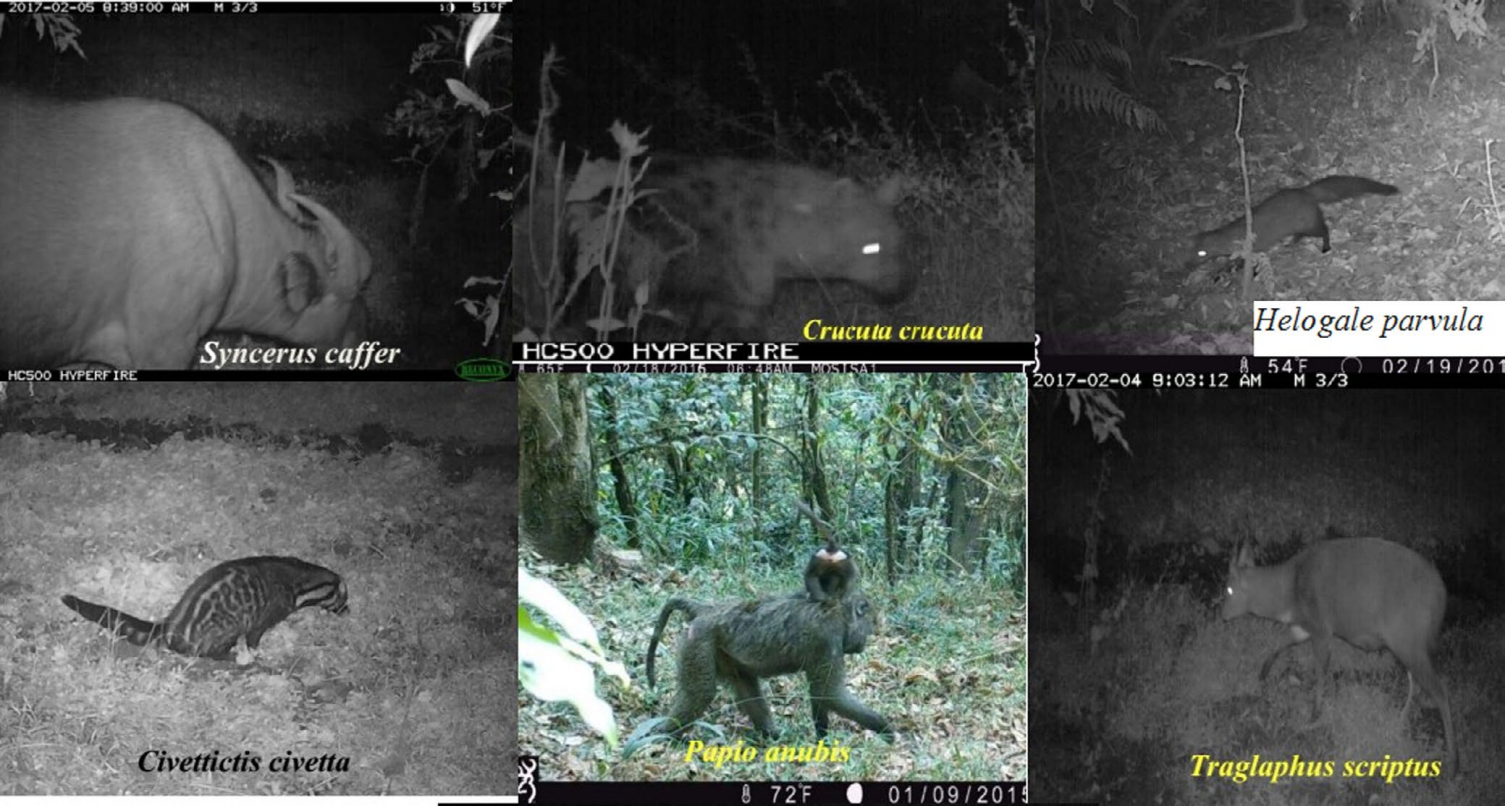
Mammalian species captured by camera trap in the Jorgo‐Wato Protected Forest

Activity periods of six mammals captured by camera trap are summarized in Table 4. The time classification of photographs captured showed that *S*. *caffer*, *C*. *crocuta*, *C*. *civetta*, and *H*. *parvula* are nocturnal, whereas *T*. *scriptus* and *P*. *anubis* are crepuscular and diurnal, respectively. *Papio anubis* was the most captured mammal by camera trap (198 photographs), followed by *S*. *caffer* (118 photographs) and *T*. *scriptus* (76 photographs). However, the activity patterns of mammals not captured by camera traps were not determined. The photographs of mammals captured by the camera trap are indicated in Figure 4.

**TABLE 4:** Activity periods of mammalian species captured by camera traps in Jorgo‐Wato Protected Forest (*N* = Total photographs)

Mammalian species	*N*	Photographic events (%)			
		Crepuscular	Nocturnal	Diurnal	Classification of activity period
*Syncerus caffer*	118	7 (5.93)	111 (94.10)	–	Nocturnal
*Traglaphus scriptus*	76	65 (85.53)	9 (11.84)	2 (2.63)	Crepuscular
*Crocuta crocuta*	56	1 (1.79)	55 (98.21)	–	Nocturnal
*Civettictis civetta*	13	–	13 (100)	–	Nocturnal
*Papio anubis*	198	–	–	198 (100)	Diurnal
*Helogale parvula*	3	–	3 (100)	–	Nocturnal

### 3.4 Habitat association of mammals

Medium and large‐sized mammals frequently utilized open (45.20%) and plantation forests (37.60%) during the wet season and open forests (38.00%), and riparian forest (27.40%) during the dry season. During both seasons, the dense forest was less frequently utilized by mammals and dense riparian forest (10.80%) during the wet season (Table 5). The distribution of signs attributed to mammal observations in different habitat types during the wet and dry seasons showed significant variation (*χ* ^2^ = 22.78, df = 3, *p* < .05).

**TABLE 5:** Habitat occurrence (%) of medium and large‐sized mammals during the wet and dry seasons in JWPF

Season	Habitat types			
	Dense forest	Open forest	Plantation forest	Riparian forest
Wet	6.80	45.20	37.60	10.80
Dry	12.60	38.00	22.00	27.40
Total	19.40	83.20	59.60	38.20

### 3.5 Behavioral response of African buffalo to anthropogenic activities

**FIGURE 5:**
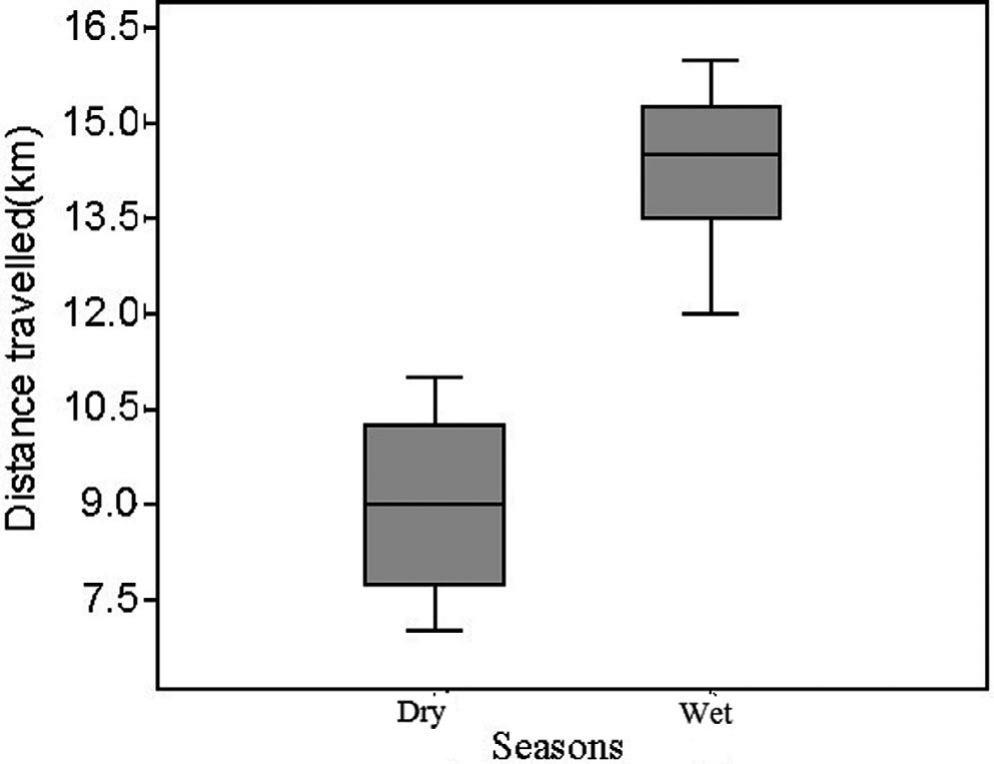
Seasonal distance travelled by African buffalo in response to anthropogenic activities in Jorgo‐Wato Protected Forest

**FIGURE 6:**
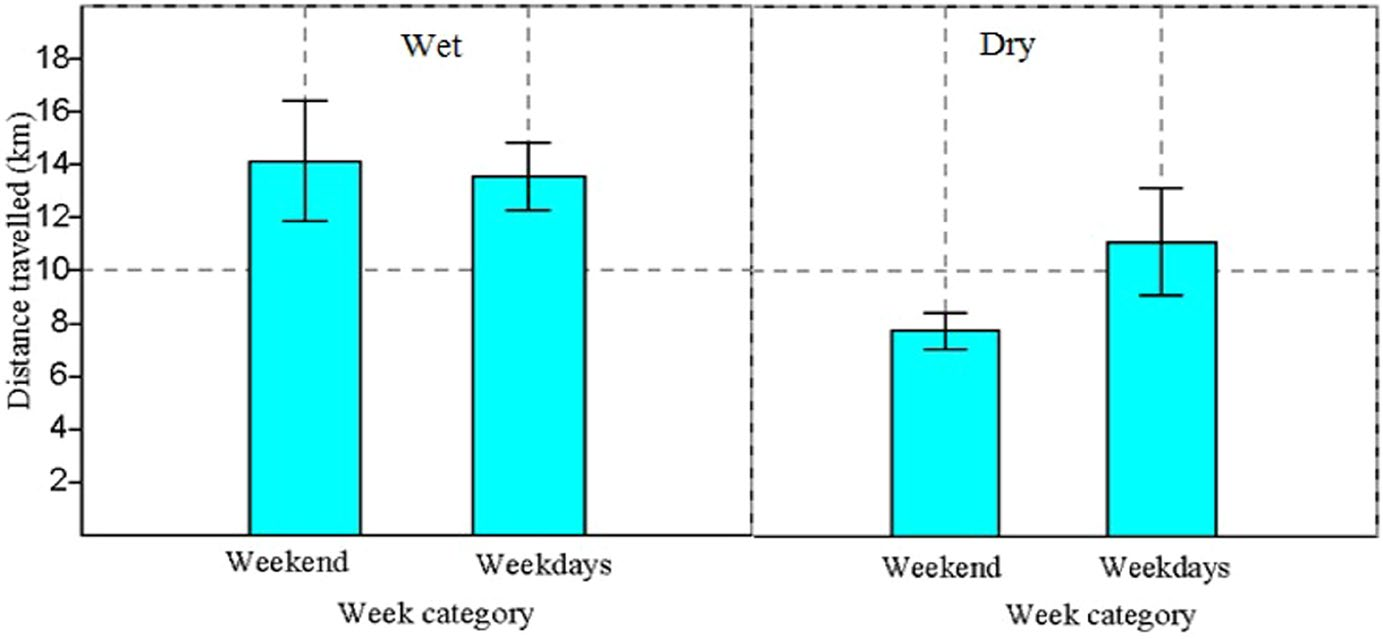
Distance travelled by African buffalo during weekdays and weekends in response to anthropogenic activities in Jorgo‐Wato Protected Forest

African buffalo traveled a longer distance during the wet season (mean = 14.33 km, SD = 1.25 km) than during the dry season (mean 9.00 km, SD = 2.16 km) (Figure 5). However, there was significant difference in the distance traveled by African buffalo during the wet and the dry seasons (*χ* ^2^ = 11.29, df = 1, *p* < .05). The mean total distance traveled by African buffalo during weekdays and weekends of the wet season was mean = 14.14 km, SD = 2.10 km, and mean = 13.57 km, SD, 1.2 km, respectively. The total distance traveled by African buffalo during weekdays and weekends of the wet season did not show a significant difference (*χ* ^2^ = 0.012, df = 1, *p* > .05). African buffalo traveled shorter distances during the weekdays (mean 7.75 km, SD = 0.66) and weekends (mean = 11. 13 km, SD = 1.90 km) in the dry season (Figure 6). However, there was no significant difference in the distance traveled between weekdays and weekends during the wet season (*χ* ^2^ = 0.979, df = 1, *p* > .05).

### 3.6 Resting preferences of African buffalo

**FIGURE 7:**
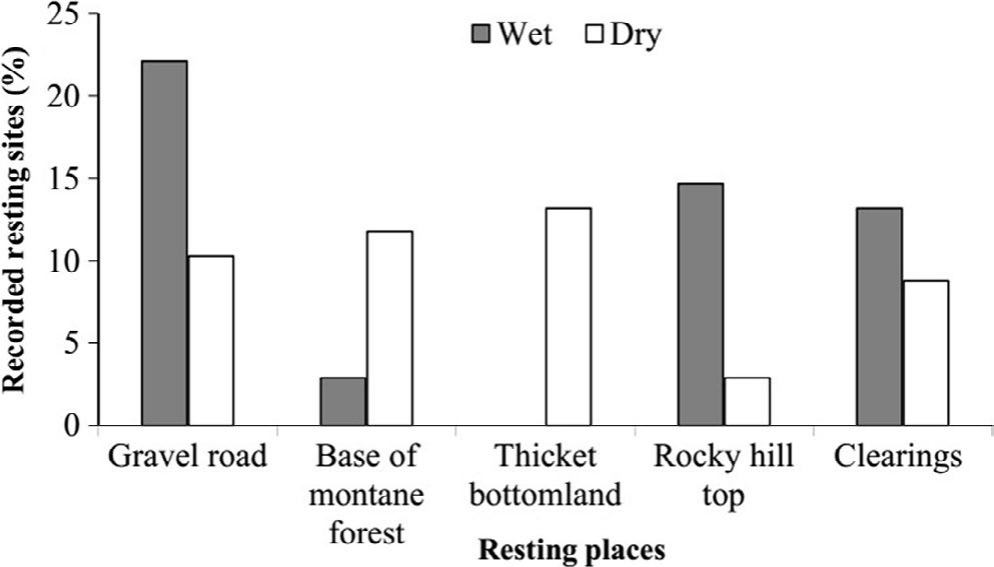
Recorded resting sites of African buffalo during the wet and dry seasons in JWPF

During the present study, a total of 402 typical resting sites (wet season = 213 and dry = 189) of African buffalo were recorded in JWPF. During the wet season, African buffalo preferred to rest on and adjacent to a gravel road (22.1%), followed by open rocky hilltops (14.7%), but not in the bottomland thicket vegetation. During the dry season, African buffalo preferred to rest in the bottomland thicket vegetation (13.2%), followed by thicket vegetation at the base of mountainous and hilly terrain (11.8%). The least resting site was recorded on rocky hilltops (2.9%) during the dry season (Figure 7). The resting sites of African buffalo recorded in different microhabitats during the wet and dry seasons were significantly different (*χ* ^2^ = 21.28, df = 4, *p* < .05).

## 4 DISCUSSION

### 4.1 Species composition of mammals

A survey of mammals in protected areas is very important to plan and determine the future conservation plan of species and their habitats. As revealed by Jackson et al. (2006), documenting the presence or absence of species of conservation concern is of great interest for ecologists to design future conservation strategies. In this study, an assessment of medium and large‐sized mammals confirmed the existence of 23 species of medium and large mammals in JWPF. Similarly, a comparable result, 28 and 19 species of mammals were reported from Dati Wolel National Park and around Wondo Genet Forest patches Ethiopia (Rabira et al., 2015). Hoverer, 15 species of mammals were recorded each from Watcha Protected Forest and Menagesha Communal Forest, Ethiopia. Variation in the number of mammal species record in the area could be due to variation in human‐induced pressure on the forest, vegetation composition, and size of the study area. However, mammalian species recorded in JWPF could be a great input for Oromia Forest and Wildlife Enterprise (OFWE) to work on the future conservation and management of wildlife in the area. To date, the conservation and management action plan of OFWE has focused only on the conservation of the forest, while the species composition of medium and large‐sized mammals' data have been not yet documented. The presence of large mammals such as *S*. *caffer* was not expected in JWPF because African buffalo subspecies were not adapted to live in dense natural and plantation forests lacking open habitats or pure grassy habitats. Moreover, the size of the forest is very small and could not be enough for large mammals requiring a large home range. In order to conserve such large mammals in the area, adjacent fallow lands not convenient for agricultural activities should be included in JWPF. JWPF is completely surrounded by the human population and threatened by various anthropogenic activities such as livestock grazing, poaching, coffee plantation, and debarking trees for beehive production. In JWPF, most mammals were recorded through indirect evidence as reported by Jiménez et al. (2010). This could be associated with the dense nature of the forest and the indicator of serious human disturbance in the forest. In areas where human disturbances are high, many mammals may shift their activity from diurnal to nocturnal or crepuscular. However, nonhuman primate species were frequently observed in the forest. This could be due to the fact that nonhuman primates are well adapted to human disturbance and disturbed habitats.

### 4.2 Relative abundance and conservation status of mammals

*Papio anubis*, *C*. *guereza*, and *C*. *aethiops* were the most abundant mammalian species in JWPF. These species are diurnal and have adapted to feed on diverse sources of food items. As reported by Johnson et al. (2012), the adaptation of these species helped them to widely distribute across different habitat types in Africa. The families of Cercopithecoidae and Colobidae are known to inhabit the forested ecosystem of long and dense trees. The distribution of these species mostly ranges from savanna grassland habitats to montane forests of Africa (Kingdon, 2003). Among the identified mammals in the study area, blue monkey and leopard are categorized under the vulnerable status of IUCN Red Data list, whereas the remaining species were categorized as least concern (IUCN, 2008). Blue monkeys are very sensitive to habitat destruction and fragmentation, and their population and range are dwindling from time to time. This could be attributed to the unlimited access of local people into protected areas for the collection of forest and nonforest products. Leopard was categorized as vulnerable probably due to reduced home range, prey, and other anthropogenic activities in the area.

### 4.3 Activity periods of selected mammals

Higher camera trap capture frequency was recorded for larger mammals such as African buffalo during the nighttime and for nonhuman primates (Olive baboon) during the daytime. This could be linked to the increased activity and home range size of large mammals. As described by Stirrat (2003), capture frequency increased as the activity, and home range size of mammals increased. Though nocturnal activity is a strategy used against predation and the advantage of utilizing underutilized food niches (Gómez et al., 2005), African buffalo restricted its activity to nighttime because of increased human activities during the daytime in JWPF. However, the high capture frequency recorded for Olive baboons could be linked to their extended group living style and large troop size. In this study, only six out of 23 mammal species were captured by a camera trap in the JWPF. In addition, only 11 out of 23 mammal species were recorded both during the wet and dry seasons. This could be ascribed to the low density of each mammal and the thick forest of JWPF (Jiménez et al., 2010). Moreover, relatively small‐sized mammals were less detected and have low capture frequency as compared to large mammals (Kelly & Holub, 2008). This could be due to the limited movement and small range use of medium mammals compared to larger mammals. Incidences of mammal records were more during the dry than the wet seasons. This could be attributed to the reduction of resources during the dry season has increased the movement of mammals in order to fulfill their nutritional requirements (Jiménez et al., 2010).

The activity period of *S*. *caffer*, *C*. *crocuta*, *C*. *civetta*, and *Helogale parvula* were confirmed to be nocturnal. However, the activity period of *P*. *anubis* was diurnal as the photographs were entirely taken during the daytime. Though photographs of all mammals were not captured by camera traps, most of the captured mammals seem to be nocturnal and crepuscular except nonhuman primates. This could be ascribed to unrestricted human access into the forest during the daytime, and poaching for bushmeat and other resource extractions. In JWPF, it seems that extensive resource extraction from the forest has forced most mammals to shift their activity periods to nocturnal and or crepuscular. For instance, the activity period of *S*. *caffer* was mainly diurnal where there is no human disturbance, but it completely shifted into nocturnal and crepuscular in JWPF.

### 4.4 Habitat association of mammals

All habitat types do not possess balanced resources and have not been equally used by ungulates throughout the year (Bjørneraas et al., 2011). Consequently, habitat utilization by ungulates varies on a seasonal and circadian basis (Demarchi & Bunnell, 1995; Dussault et al., 2004) and is governed by trade‐offs between associated costs and benefits (Rettie & Messier, 2000). To optimize the cost and benefits, ungulates use different habitats at different periods. In the present study, mammals avoid open forest habitats during the daytime due to high human disturbances in the forest. Consequently, they utilized open habitats at night due to the high quality and quantity of forages in open forest habitats as described by Hebblewhite et al. (2008) and Godvik et al. (2009). As reported by Melletti et al. (2007), for instance, forest buffalo were highly dependent on open forest habitats and clearings in Bai‐Hokou forests of Dzanga‐Ndoki National Park, Central African Republic. In the present study, mammals also preferred plantation and open forests during the wet season due to the availability of adequate forages and limited human access. Large mammals such as African buffalo experience high heat stress in open savanna habitats and restrict themselves from daylight foraging (Prins, 1996). However, large mammals such as African buffalo have shifted their foraging period into nocturnal and or crepuscular mainly due to high human disturbances in JWPF as reported by Di Bitetti et al. (2008). Di Bitetti et al. (2008) also stated that increased poaching pressure can alter activity patterns of hunted species into nocturnal. As reported by Skinner and Smithers (1990), African buffalo graze closer to rivers and take shelter in the thick riverine vegetation during the dry season. Hence, the difference in habitat association of mammals in the present study area might be mainly a functional response to anthropogenic activities in the forest. An increased habitat association of mammals to riparian habitats could be linked to risk avoidance in the open forest. To optimize cost–benefit relationships, ungulates may use habitats with good cover during the daytime (Demarchi & Bunnell, 1995; Dussault et al., 2004) and visit open forage‐rich habitats during the night as they are less visible to humans (Godvik et al., 2009; Lykkja et al., 2009). In the present study, mammals did not prefer dense forest due to reduced forages as reported by Perrin and Brereton‐Stiles (1999).

### 4.5 Behavioral response of African buffalo to anthropogenic activities

African buffalo traveled a longer distance during the wet season compared to the dry season. This could be due to the fact that the local community is less likely to go to the forest for resource extraction during the wet season as they are always busy with agricultural activities. However, the dry season is the resting period for the local people due to low agricultural activities. Consequently, the local people frequently visit the forest for resources extraction and bushmeat hunting thereby limit the movement of large and shy mammals such as African buffalo to their refugia. Moreover, African buffalo relatively traveled longer distances during weekdays and weekends of the wet season but low during the same periods of the dry season. The long resting time during the dry weekdays and weekend provides the local people an opportunity to frequently visit the forest. This could limit the activity of mammals to their refugia during the daytime. However, during the wet season, the local people are fully engaged in agricultural activities and are less likely to visit the forest for resource extraction. As described by Nix et al. (2018), the movement of animals is very slow in areas where human activities are high. In such areas, animals use contrasting activity periods with humans to avoid risks. Animals travel longer distances and spent more time away from their refugia in areas where human disturbance is low or absent (Perona et al., 2019).

### 4.6 Resting preferences of African buffalo

The present study has revealed that buffalo preferred to rest on gravel roads, open rocky hilltops, and clearings during the wet season. These resting sites were used during the night as these sites were frequently visited by humans during daylight hours. Such open microhabitat preferences of buffalo could facilitate social interaction and rumination processes of members watching dangers from a distance. The inclination of African buffalo to sleep on gravel roads at night indicated a strong desire for open habitats and/or a scarcity of open habitats and glades in JWPF. This goes in line with the findings of Senft et al. (1985) and Bailey (2004), who reported that open glades and open wooded forests with short grass cover and good visibility are generally preferred by herbivores for resting and nonforaging activities such as rumination. Moreover, it could facilitate social interactions such as grooming among members of the herd (Melletti et al., 2007). During the nighttime, the movement of buffalo from dense forest, thicket, and moist vegetation (where they spent much of their daytime hours) into open forests, gravel roads, and clearings could be ascribed to foraging and ventilation of the body. Studies showed that mammal's shelter under trees during the rainy time but move out into open habitats when it stops for ventilation (Fonkwo et al., 2011; Obioha et al., 2012). Contrary to Jorgo‐Wato buffalo, forest buffalo rest in the forest at night and in clearings during the day (Melletti et al., 2007). Consequently, they might have been more susceptible to poachers. However, Jorgo‐Wato buffalo preferred to rest more in the thicket vegetation and at the base of montane forests and hilly terrain during the daytime. This could be attributed to the avoidance of risks encountered in clearings and open forests. Buffalo's preference for mountainous terrain and rocky hilltops during the wet season could be linked to thermoregulation as revealed by Harris et al. (2002). As stated by Harris et al. (2002), areas of high ground are warmer than bottomland at night and hence preferred for nocturnal resting by herbivores.

## 5 CONCLUSION

JWPF is a potential wildlife habitat for diverse arrays of medium and large mammals in the western parts of Ethiopia. However, studies on JWPF in general and mammals, in particular, have not yet been well documented because of the inaccessibility and remoteness of the area. In this study, a combination of field survey techniques such as camera traps, direct observation, and indirect evidence was employed to document the medium and large mammal species composition of JWPF. A total of 23 species of medium and large mammals were recorded in the study area during the wet and dry seasons. Most of these mammals were recorded by camera traps and indirect evidence because they were rarely observed due to human disturbances. The unlimited human access and resource extraction in the JWPF have made direct observation of mammals difficult except nonhuman primates. The activity of other mammals in JWPF was confined to the nighttime due to high anthropogenic activities in the forest. Though JWPF is legally protected as one of the top forest priority areas in Ethiopia, it is seriously threatened by anthropogenic activities. This study also confirms the significance of JWPF for wider ranges of biodiversity conservation and the need for a further plan for the protection, conservation, and management of wildlife species in the area.

## CONFLICT OF INTEREST

There are no conflicting interests.

## AUTHOR CONTRIBUTION

**Mosissa Geleta Erena:** Conceptualization (lead); data curation (lead); formal analysis (lead); funding acquisition (lead); investigation (lead); methodology (lead); project administration (lead); resources (lead); software (lead); supervision (lead); validation (lead); visualization (lead); writing – original draft (lead); writing – review and editing (lead).

## Data Availability

Data used in this work are archived in Dryad Digital Repository, https://doi.org/10.5061/dryad.6hdr7sr2g.
